# Performance Study of a Torsional Wave Sensor and Cervical Tissue Characterization

**DOI:** 10.3390/s17092078

**Published:** 2017-09-11

**Authors:** Antonio Callejas, Antonio Gomez, Juan Melchor, Miguel Riveiro, Paloma Massó, Jorge Torres, Modesto T. López-López, Guillermo Rus

**Affiliations:** 1 Department of Structural Mechanics, University of Granada, 18071 Granada , Spain; acallejas@ugr.es (A.C.); mriveiro@ugr.es (M.R.); pmasso@ugr.es (P.M.); jtp31@correo.ugr.es (J.T.); grus@ugr.es (G.R.); 2 Department of Mechanical Engineering, University College London, WC1E 7JE London, UK; aj.gomez@ucl.ac.uk; 3 Biosanitary Research Institute, 18012 Granada, Spain; 4 Department of Applied Physics, University of Granada, 18071 Granada, Spain; modesto@ugr.es

**Keywords:** torsional wave sensor, tissue mimicking phantom, cervical tissue, rheological model, rheometry experiment, sensitivity study, complex shear modulus

## Abstract

A novel torsional wave sensor designed to characterize mechanical properties of soft tissues is presented in this work. Elastography is a widely used technique since the 1990s to map tissue stiffness. Moreover, quantitative elastography uses the velocity of shear waves to achieve the shear stiffness. This technique exhibits significant limitations caused by the difficulty of the separation between longitudinal and shear waves and the pressure applied while measuring. To overcome these drawbacks, the proposed torsional wave sensor can isolate a pure shear wave, avoiding the possibility of multiple wave interference. It comprises a rotational actuator disk and a piezoceramic receiver ring circumferentially aligned. Both allow the transmission of shear waves that interact with the tissue before being received. Experimental tests are performed using tissue mimicking phantoms and cervical tissues. One contribution is a sensor sensitivity study that has been conducted to evaluate the robustness of the new proposed torsional wave elastography (TWE) technique. The variables object of the study are both the applied pressure and the angle of incidence sensor–phantom. The other contribution consists of a cervical tissue characterization. To this end, three rheological models have fit the experimental data and a static independent testing method has been performed. The proposed methodology permits the reconstruction of the mechanical constants from the propagated shear wave, providing a proof of principle and warranting further studies to confirm the validity of the results.

## 1. Introduction

Several authors have suggested that the mechanical functionality of soft tissue is a highly relevant clinical parameter to a broad range of pathologies. For example, Berghella [1] hypothesized that the elasticity of the cervical tissue below typical values conditioned delayed term birth and failure of the required delivery induction, which is the leading cause of fetal suffering. To this end, in the last years, measuring shear viscoelastic constants in soft tissues has become a challenge. There are existing commercial shear sensors available, although some limitations, like the upper threshold of detectable stiffness, have been reported [2].

In this field, two main elastography techniques have been developed for the measurement of tissue stiffness: Static elastography (SE) and dynamic elastography (DE). On one hand, SE provides strain maps. The tissue is manually compressed with the probe until no further tissue deformation is observed in the B-mode image (maximum deformation). Then, the maximum deformability of the cervix is quantified as the ratio of the anterior-posterior distance (thickness), before (reference configuration) and after compression application [3]. A qualitative description of the relative strain distribution is obtained, which is not a quantitative description of the real stiffness of the tissue. Despite the simplicity and compatibility of the technique with standard equipment, measurements are highly dependent on the pressure applied by the practitioner. The lack of a standard is currently one of the challenges for the clinical use of SE [4,5,6].

On the other hand, DE techniques are based on shear waves propagation and provide a quantitative description of tissue stiffness compared to static methods [7,8,9,10]. A great advantage of some of these techniques is the ability to depict the spatial distribution of mechanical properties of the biological tissues [2]. However, some limitations have been identified such as limitation of the acquisition frame rate to recover an entire image of the medium, and the deposited energy in the medium [11,12].

Recently, a new family of sensors based on torsional waves has been patented, such as the US 5321333 A [13], which belongs to the field of low frequency sensors for the generation or reception of torsional shear waves in a substantially solid medium. This new technique solves the problem of the strong attenuation of induced shear waves, providing a new way for the development of new noninvasive tissue characterization techniques [14]. In this work, a new torsional wave sensor, designed and manufactured by our group, has been used [15]. The probe used in this work has been developed to provide quick measurements of the shear stiffness of the cervix. It has been optimized reducing the spurious waves contamination (P-waves), using a similar procedure as depicted in [15], the excitation energy deposited in the tissue and the dependence with the applied pressure sensor-tissue. The simplicity of the method ensures an easy clinical procedure and a short learning curve for the operator. Although further investigations are required, the work hypothesis considers that the averaged and local measurement of shear stiffness might be sufficiently significant for characterizing the elastic condition of the cervix.

Compared with other DE techniques such as SSI (SuperSonic Imagine, Aix-en-Provence, France) [2,16], the torsional wave elastography (TWE) offers a higher range of frequencies, up to 10 kHz, due to a high acquisition rate up to 100,000 fps compared with the 6000 fps of the SSI technique. This provides a broader spectrum of the mechanical behavior of the cervical tissue, which yields a finer viscoelastic characterization. At the same time, this high acquisition rate allows the analysis of perturbations of higher order by obtaining more data points per wavelength. Another advantage of the TWE technique is based on the lower energy deposited in the tissue during the procedure. The low energy delivery favors a high repeatability of the measurements, which, combined with the capacity of testing different pressure level scenarios, might minimize the inter and intra-operator deviations.

In the field of elasticity imaging, quantifying viscoelastic properties in soft tissues is a challenging problem. To date, several rheological models have been used to characterize different soft tissues and tissue mimicking phantoms. The Kelvin–Voigt (KV) model has been used specifically to quantify viscoelastic properties in soft tissues. Apart from this method, for characterization of the viscoelastic media, some authors have used the Maxwell (M), generalized Maxwell (GM) model, the Zener (Z) model and the Kelvin–Voigt fractional derivative (KVFD) model [17,18,19,20,21,22,23,24]. Nowadays, little is known about the mechanical behavior of cervical tissue. Genisson et al. [25] have shown that the pathological cervix seems to be a softer than normal cervix. A study in pregnant ewes carried out by Peralta et al. [26] indicates that stiffness of the uterine cervix changes throughout the maturation process. Miklos et al. in a study performed on 24 patients under small compressions, and a range of frequencies from 0.1 to 100 Hz [27] concluded that normal cervical tissue exhibited values of complex modulus over the same range. Finally, Jiang et al. applied 3D multifrequency MR elastography (3DMMRE) to study the viscoelasticity of the uterus and cervix, showing sensitivity to structural and functional changes of the endometrium and myometrium during the menstrual cycle [28].

As it was stated in this paper, we propose a novel torsional wave sensor capable of emitting and receiving shear waves. This sensor can measure the shear wave speed in tissue mimicking phantoms and soft tissues. The structure of the sensor probe is described in detail, with an optimal design necessary to avoid the resonant frequency in the emitter and the receiver. Then, the experimental setup and protocol are presented. Experimental tests of the mechanical properties of the tissue mimicking phantoms were performed to confirm that the measurements are independent of the applied pressure and the angle between probe and phantom surface. Finally, a human cervical tissue characterization study is carried out. Ex vivo samples have been tested and three rheological models have been studied to understand the behavior of cervical tissues under shear wave propagation. Finally, an alternative testing method based on measuring viscoelastic parameters is used for comparison with the TWE technique.

## 2. Materials and Methods

### 2.1. Structure of the Sensor

**Figure 1:**
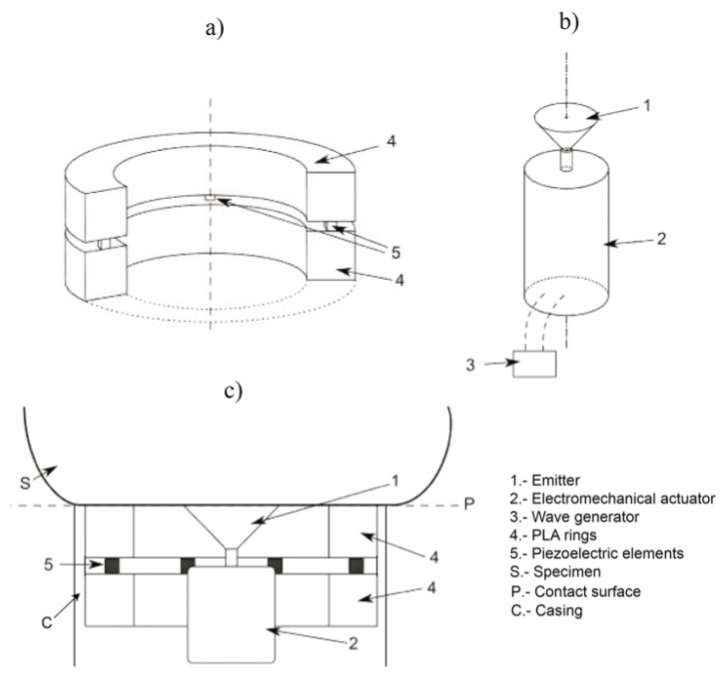
Schematic view of the sensor elements. (**a**) the receiver; (**b**) the emitter; (**c**) contact sensor–phantom.

The torsional device is composed of an emitter, a receiver and a casing that holds both. The emitter, responsible for the transmission of the wave which travels through the phantom, consists of a PLA (polylactic acid) disk, printed in 3D, whose rotational movement is due to an electromechanical actuator (see Figure 1b). This actuator is covered by a Faraday’s cage of aluminum paper to nullify any effect of external electromagnetic fields. To improve the attachment between the aluminum and the actuator, a silver conductive epoxy, used as adhesive that increases the conductivity, is used. The actuator is excited electrically by a wave generator, where some parameters like frequency, voltage or working cycle are controlled.

The receiver, showed in Figure 1a, is formed by two PLA rings with four slots in the inner face of the ring, where the four ceramic piezoelectric elements, whose material is NCE51, are fitted. All the piezoelectric elements are connected to each other with a copper rod, which works as a bracket too. The rings mentioned above provide the inertia to reduce the resonant frequency and the stiffness to reduce dilatational waves. The array of transversely-polarized piezoceramic elements is connected to the ring by silver conductive epoxy. The piezoceramic elements are responsible for transforming the mechanical movement into an electric signal. Thus, each piezoelectric element is in contact with two electrodes of different charges.

The third part is a casing with geometrical and material selection to control the mechanical cross-talk and hold the emitter and the receiver (see Figure 1c). This configuration eliminates the masking p-waves that systematically arises at the boundaries of the regular contact or comb transducers [29].

### 2.2. Simplified Analytical Model

To respond to the principles of an optimal design of a torsional wave sensor considering the extraction of biomechanical properties, it is necessary to compute Finite Element Models (FEM) as a first step [15,29,30]. Thus, the resonant frequency can be determined for torsional waves for the emitter and the receiver using Finite Element Analysis Program (FEAP) software [31]. Therefore, a simplified model with a disk as a transmitter and a ring as a receiver (to facilitate the accessibility of experimental measurements) was developed to validate the output of resonant frequency of FEM.

The fundamentals of a simplified analytical model for an oscillatory movement of a torsional sensor are shown as follows. Consequently, the piezoelectricity coupling is introduced as a strain law uniformly distributed and directly proportional to the electrical field [32].

A single degree of freedom system is established, where the eigenproblem is reduced to a single frequency and a single mode. The movement is assumed to be subjected to the torsion rotation Θ in radians. In addition, the dynamic equilibrium of torsional moment is defined as:

(1) $$K\Theta +I\frac{d^{2}\Theta }{dt^{2}}=0,$$

where *K* is the stiffness coefficient measured in [Nm/rad] and *I* is the inertia moment. The steady-state solution has the following expression:

(2) $$\Theta =\Theta^{0}\sin(\omega t),$$

where ω is the natural frequency valid for the equilibrium Equation (1).

Considering the subsystem eigenfrequency in the case of cylinder mass and analyzing the relationship between the equations of equilibrium and the moment of inertia for a cylinder, the frequency obtained is derived as

(3) $$f=\frac{1}{2\pi }\sqrt{\frac{nabd^{2}\mu }{\frac{\pi }{2}l^{\mathrm{eff}}hr^{4}\rho }}.$$

Subsequently, in the case of ring mass, analyzing the subsystem eigenfrequency with the same equilibrium equation and the moment of inertia of a ring shape, we extract the next frequency

(4) $$f=\frac{1}{2\pi }\sqrt{\frac{nabd^{2}\mu }{2\pi l^{\mathrm{eff}}hmr^{3}\rho }},$$

where *n* is the number of piezoelectric elements, *a* and *b* are the plane dimensions of the piezoelectric ceramic, *d* the distance from the center of rotation, μ the shear modulus, $l^{\mathrm{eff}}$ the effective length between piezoelectric ceramics, *h* the height of the cylinder or the ring, *r* the radius of the cylinder or the ring, *m* the thickness of the ring in the radial direction and ρ the density of the mass blocks.

The sensor is designed with a cylinder mass and a ring mass for each inertia subsystems of emission and reception. Then, their eigenfrequencies are fitted to optimize the resonant amplification.

Finally, the frequency of resonance obtained from FEM simulation is analyzed measuring the time between peaks of cycles. The result coincides with the resonance frequencies of the transducer described in the Table 1 with a percentage of error below 1% in each case.

### 2.3. Experimental Setup

**Figure 2:**
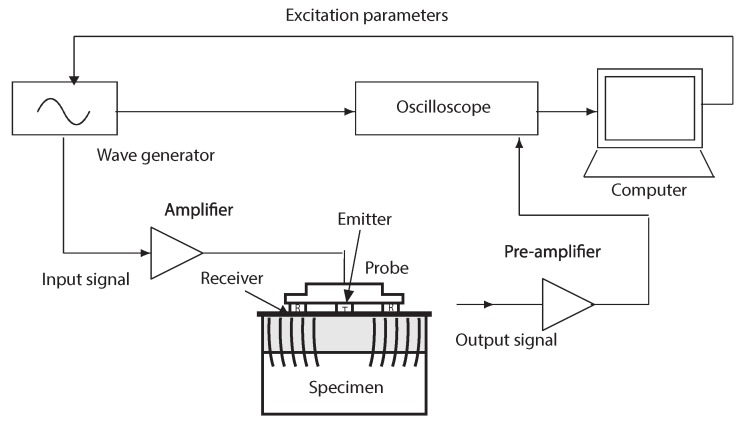
Experimental configuration of the excitation–propagation measurement system.

The gelatin gels and cervical tissues were excited by a low-frequency sine-burst at different frequencies (from 300 Hz to 1000 Hz). Excitation signals were generated by an arbitrary wave generator (Agilent 33220A, Santa Clara, CA, United States) and amplified (Radio Frequency Power Amplifier 150 A, 150 W, 100 MHz) before reaching the disk emitter. Measurements were acquired for 16 V mechanical actuator voltage amplitude that provides particle displacements at the emitter surface. The transmitted shear wave motion was propagated across the homogeneous phantoms and recorded by the receiver. Pre-amplification of received signal (Olympus, 576, 172 × 42.5 dB, Waltham, MA, United States) and low-pass filtering is necessary for recording a signal of greater amplitude than the signal noise level. The wave generator is synchronized with the oscilloscope (HDO 4034, 350 MHz, 2.5 GS/s, Santa Clara, CA, United States ) to record the start of the signal. As a final step, a Matlab (Release 2014b, MathWorks, Natick, United States) optimization algorithm yields the shear wave speed. The experimental setup of the specimens is shown in Figure 2.

### 2.4. Specimens and Experimental Protocols

#### 2.4.1. Sensitivity Study

Gelatin samples from porcine skin (Type A, 300 Bloom, G2500, Sigma-Aldrich, St. Louis, MO, USA) were constructed to test the sensor sensitivity under different applied pressures, sensor–specimens and different angles of incidence. Six homogeneous phantoms with 8% and 10% (*w*/*w*) gelatine concentrations were tested. Gelatine powder and purified water at 90 ${}^{\circ }$C were stirred for 10 min to ensure gelatine dissolution. Molten gelatine was cast in cylindrical molds (6.8 cm diameter, 1.35 cm height) and kept at room temperature for 2 h before being stored in the refrigerator at 5 ${}^{\circ }$C. The phantoms were taken out one day after being stored in the fridge and were tested when they reached the laboratory temperature (22 ± 1 ${}^{\circ }$C).

**Figure 3:**
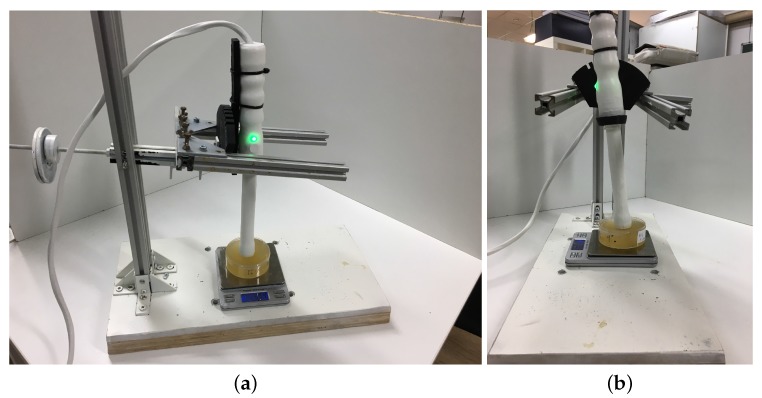
A counterweight device to control the applied pressure (phantom positioned on a balance) and the angle of incidence phantom-sensor. (**a**) angle of incidence 0${}^{\circ }$; (**b**) angle of incidence 7.5${}^{\circ }$.

All measurements were carried out with the same experimental protocol. A counterweight device was specifically designed to control the applied pressure and the angle of incidence phantom surface–sensor (see Figure 3). The phantoms were excited with a range of frequencies from 300 to 1000 Hz to avoid resonance phenomena in the rings that mimic shear waves. The phantoms were positioned at the balance to quantify the applied force during the measurement. Measurements from four applied pressures (from 4.81 to 24.06 kPa) and two angles of incidence phantom surface-sensor (0 and 7.5${}^{\circ }$) were performed. The material density used was 1000 $\mathrm{kg}/m^{3}$.

#### 2.4.2. Cervical Tissue Characterization

**Figure 4:**
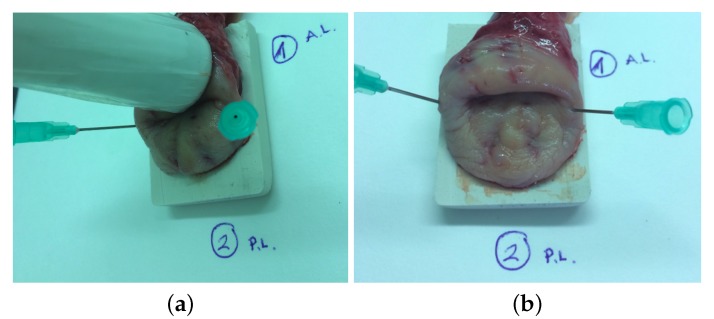
(**a**) measurement with the torsional wave sensor of a cervical tissue sample; (**b**) cervical tissue sample.

To obtain the viscoelastic properties, a test ex vivo on human cervical tissue samples from 3 healthy women aged from 55 to 74 years old were carried out using TWE and rheometry techniques (see Figure 4). Patient consent (the study was conducted according to the Declaration of Helsinki Principles and the agreement of the ethical committees of the University of Granada and the Universitary Hospital Complex of Granada) was obtained before carrying out the measurements. All patients were diagnosed with uterine prolapse (II and III grade) that required a partial or complete hysterectomy. The surgical procedures were performed at the San Cecilio University Hospital, Granada (Spain). The organs were placed in Phosphate Buffered Saline (PBS) and transferred to the Pathological Anatomy Laboratory in a refrigerator. The specimens were tested with the torsional wave sensor before being excised for rheometry experiments. Two pins were placed along the contours to prevent distortion of the tissue, and the cervix was placed on top of a rectangular piece of sound-absorbing rubber. Both measurements were performed at room temperature and typically within 2.5 h after being transferred to the laboratory, measuring three times each sample to obtain the mean and standard deviation. Given the independence of the applied pressure and the angle of incidence sensor-specimen in the sensitivity study, the cervical tissue samples were tested with the torsional wave sensor using the same range of frequencies (from 300 to 1000 Hz). After that experiment, circumferential samples were cut by a pathologist for rheometry experiments, 20 mm in diameter and 3–5 mm thick.

### 2.5. Time of Flight—Signal Processing

Time of flight calculation is conceptually simple, although it is necessary to introduce simplifying hypotheses. Physically, S-type waves are originated by the actuator and are transmitted through the specimen to the piezoelectric sensor, where they produce the deformation thereof and, consequently, an electric potential capturable by an oscilloscope.

In a purely theoretical approach, the signal in the sensor channel has null amplitude until the shear wave reaches the sensor producing a non-zero amplitude response. In practice, the presence of undesired interference signals from multiple sources requires a measurement and processing protocol to improve the quality of the resultant signal.

The signal processing includes the average of multiple measurements to decrease noise. Thereby, multiple identical measurements spaced by a relaxation time to completely dissipate previous waves were used. In addition, a low-pass filter in frequency domain with a cutoff frequency of 8000 Hz was applied to eliminate distortions generated from the electronics that controls the equipment.

Averaging and filters did not completely eliminate the electromagnetic coupling between emission and reception channels. This situation means that the received signal had an instant response time. Several tests suggest that the electromagnetic coupling component depends, on first approximation, only on the emitted signal power and in certain cases on external environmental components, like a remarkable increase of the humidity. In this work, the “calibration” measurement (without contact with the specimen) and the measurement of the specimen had the same coupling component. Thus, subtracting the calibration measurement from the measured signal, this effect can be eliminated.

The analysis of the resultant signal allows obtaining a first time of flight time measurement as the difference between the starting time of the received signal and the starting time of the excitation signal. Certain simplifications must be assumed for this analysis since there are phenomena such as imperfect centering of the transmitter with respect to the four piezoelectric receivers and transient effects associated with the engine start, high frequencies transmission and other effects whose quantification are beyond the scope of this work. Taking this into account, several approaches have been analyzed.

1. To normalize the signal with the first maximum (in absolute value) and to calculate the start from this maximum, using a reference value (typically 5% or 0.05).
2. To normalize the signal with the first maximum (in absolute value) and to calculate the start from the crossing of the horizontal axis with the tangent on the linear zone of the first sinusoidal section of the curve.
3. To calculate the frequency using the first two maximum and minimum and to extend a quarter of a cycle from the first function extremum.
4. To generate a parameter-dependent reference signal that simulates the expected signal. By means of an inverse problem (a combination of genetic algorithms and quasi-Newton type optimization algorithms), to perform and to adjust the experimental and the reference signal minimizing a residue from weight functions.

In this work, the last approach was used.

### 2.6. Shear Speed from Time of Flight

Ideally, the shear velocity can be calculated directly from the time of flight

(5) $$Velocity=\frac{Distance}{Time\;\;of\;\;flight}.$$

However, the time of flight measured directly from the sensor signal is not the correct wave time of flight time because there is a delay associated with multiple effects (transmission in the PLA, delays related to electronics and piezoelectric devices...). These types of effects can be combined into a single delay time factor, so the Equation (5) results in

(6) $$Velocity=\frac{Distance}{Time\;\;of\;\;flight-delay}.$$

This internal delay is characteristic of the measurement configuration and must be estimated previously. Assuming that the internal delay depends only on the material and the frequency excitation, time of flight of measurements at different distance emitter-receiver can be extended to zero distance obtaining a non-zero value of time that is the delay searched.

### 2.7. Rheological Models

**Figure 5:**
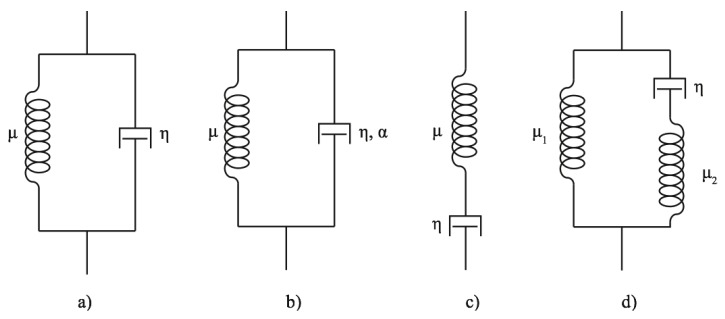
Rheological models. (**a**) the Kelvin–Voigt model; (**b**) the Kelvin–Voigt Fractional Derivative model; (**c**) the Maxwell model; (**d**) the Zener model.

Similarly to many soft tissues, the behavior of cervical tissue can be modeled as linear viscoelastic media and several models have been already studied [17,33,34]. Here, we propose the Kelvin–Voigt, Kelvin–Voigt Fractional Derivative, Maxwell and Zener models, which are the most widely used (see Figure 5).

The viscoelastic modulus ($G^{*}$) (Equation (7)), which is composed of a real part ($G^{\prime }$), elastic or storage modulus and an imaginary part ($G^{{}^{\prime \prime }}$), viscous or loss modulus, characterizes the viscoelasticity of the material [35]:

(7) $$G^{*}=G^{{}^{\prime }}\left(\omega \right)+iG^{{}^{\prime \prime }}\left(\omega \right).$$

The following rheological models relate the elasticity and viscosity by the complex shear modulus.

The Maxwell model (M) can be represented by a spring and a dashpot in series, as shown in the Figure 5. The complex shear moduli for this model is

(8) $$G^{*M}\left(\omega \right)=\frac{\mu \eta \omega^{2}}{\mu^{2}+\omega^{2}\eta^{2}}+i\frac{\mu^{2}\eta \omega }{\mu^{2}+\omega^{2}\eta^{2}}.$$

The Kelvin–Voigt (KV) rheological model is characterized by elastic μ and viscous η coefficients. This rheological model consists of a spring and dashpot placed in parallel, which describes tissues as solids that can creep but show little stress relaxation. The corresponding complex shear moduli for the KV model is

(9) $$G^{*KV}\left(\omega \right)=\mu_{1}+i\omega \eta ,$$

where $\mu_{1}$ is the shear elasticity (in Pa) and η is the shear viscosity (in Pa · s).

The Kelvin–Voigt Fractional Derivative model (KVFD), obtained by a generalization of the Kelvin–Voigt model, is a three-parameter fractional-order model. The first derivative in time in the KV model is replaced by a fractional-order derivative of order α [36]. The complex shear moduli for the KVFD model is [37,38]

(10) $$G^{*KVFD}\left(\omega \right)=\mu_{1}+\eta \omega^{\alpha }cos\left(\frac{\alpha \pi }{2}\right)+i\eta ,\omega^{\alpha }sin\left(\frac{\alpha \pi }{2}\right),$$

where α is the fractional derivative power (0≤α≤1).

The Zener model (Z) expresses both the stress relaxation and the creep in linear viscoelastic polymeric solids [39]. The complex shear modulus for the Zener model in the frequency response is given by [24]

(11) $$G^{*Z}\left(\omega \right)=\frac{\mu_{1}\mu_{2}^{2}+\omega^{2}\eta^{2}\left(\mu_{1}+\mu_{2}\right)}{\mu_{2}^{2}+\omega^{2}\eta^{2}}+i\frac{\mu_{2}^{2}\eta \omega }{\mu_{2}^{2}+\omega^{2}\eta^{2}},$$

where $\mu_{1}$ and $\mu_{2}$ are the elasticities (in Pa) and η is the shear viscosity (in Pa · s).

Dispersion curves are plots of shear wave speed ($c_{s}$) as a function of angular frequency ω. For mass density ρ, the relationship between $c_{s}$ and the complex shear modulus is well known to be [40]

(12) $$c_{s}\left(\omega \right)=\sqrt{\frac{2(G^{\prime 2}+G^{\prime \prime 2})}{\rho (G^{\prime }+\sqrt{G^{\prime 2}+G^{\prime \prime 2}})}}.$$

### 2.8. Rheometry Experiments

**Figure 6:**
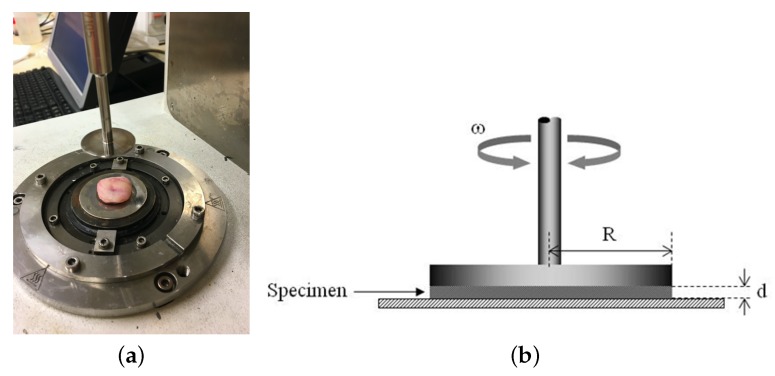
(**a**) A controlled-rate magnetorheometer MCR 300 Physica-Anton Paar, Graz, Austria. Pink matter corresponds to the cervical tissue sample. (**b**) schematic view of the rheometer.

Rheometry is a technique that studies the relationship between stress–strain in materials that are capable of flowing, thus allowing to get mechanical properties related to elasticity and viscosity, which usually leads to the development of a constitutive relation. In this work, these properties were evaluated under shear stress with a controlled-rate magnetorheometer (MCR 300 Physica-Anton Paar, Graz, Austria) (see Figure 6). As inertia of the sample was negligible in oscillatory shear deformation, it required that a small phantom thickness compared with the wavelength of shear waves propagated through the medium at the frequency of oscillation [41]. The measuring system geometry was a 20 mm diameter parallel plate set for a gap width dependent on the sample thickness, as a rule 3–5 mm. 

All measurements were conducted at room temperature to avoid loss of consistency. The experiment started with a squeezing compression of the rotor plate, no less than 0.2 N, for preventing slippery conditions. To ensure the reproducibility of the measurements, a pre-shear (10 cycles at 1 Hz) was applied with a waiting time of 10 s before measuring. The samples were subjected to a sinusoidal shear stress and the corresponding shear strain was measured, according to the constitutive equation for linear viscoelastic materials where

(13) $$\sigma =\sigma_{0}ei\omega t,$$

(14) $$\gamma =\gamma_{0}e^{i(\omega t-\delta ),}$$

are, respectively, the shear stress and strain, $\sigma_{0}$ is the stress amplitude, $\gamma_{0}$ the strain amplitude, ω the angular frequency, and δ the stress–strain phase lag. Two oscillatory tests were planned to obtain the viscoelastic modulus ($G^{*}$).

The experiments were [42,43]: (i) amplitude sweep: frequency was kept constant at 1, 2, 5, 10 and 20 Hz, and shear amplitude was varied up to values in which nonlinear behavior was reached. Nonlinearity was developed and was clearly distinguishable when $G^{{}^{\prime }}$ and $G^{{}^{\prime \prime }}$ were not only functions of ω but also strongly depended on $\gamma_{0}$. The frequency range was not higher owing to the linearity of oscillations that can not be ensured when the rotor (upper plate) and the sample inertia effects become significant at high frequencies [41]; and (ii) frequency sweep: shear amplitude was kept constant into the viscoelastic limit region, determined by the previous test and the frequency was swept from 0.5 Hz to 100 Hz logarithmically to obtain the oscillograms ($G^{{}^{\prime }}$ and $G^{{}^{\prime \prime }}$ as a function of ω).

### 2.9. Viscoelastic Parameters Reconstruction

The characteristic parameters of a viscoelastic material were obtained fitting the experimental results with the rheological models indicated in the previous section. Two different techniques (rheometry and TWE) were used in each sample of cervical tissue. For each sample and methodology, velocities at several frequencies were calculated and the mean of the velocities over all the measurements were used for further analysis.

In the rheometry study, the real and the imaginary parts of the viscoelastic modulus were obtained from the rheometer and the shear wave speed was calculated directly using Equations (7) and (12). For the torsional wave sensor, the shear wave speeds were obtained following the approach depicted in Section 2.5.

This allowed the construction of a speed-frequency chart for each tissue sample. In the next section will be represented the mean and standard deviation of the shear wave speeds using data from all the tissue samples. The high differences on the frequency ranges of the two techniques should be noted.

Every set of measurements was fitted to the models to obtain the parameter values. This adjustment was made by means of an inverse problem using a combination of genetic algorithms and quasi-Newton type optimization algorithms.

Three approaches were used and represented varying the fitted data using (a) shear wave speeds exclusively from rheometry, (b) speeds exclusively from the torsional sensor, and (c) all of the available data. The fitting procedure was applied to each tissue sample to obtain the average adjustment curves (including standard deviations). Two options were available: using the average values of the parameters to generate average curves or directly averaging the values of the curves. It should be highlighted that these adjustments are not equal since the models were nonlinear. The first approach was used in this work.

## 3. Results

**Figure 7:**
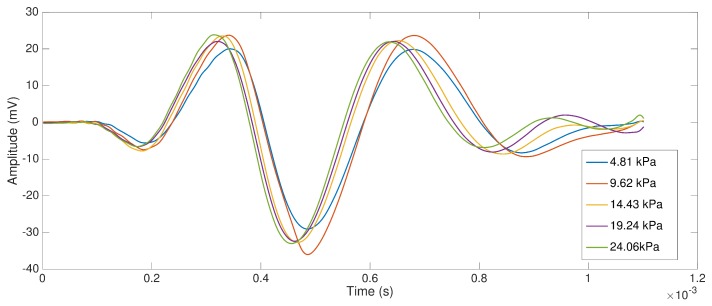
Shear wave signals at different pressures’ phantom–sensors (from 4.81 to 24.06 kPa), frequency: 300 Hz; gelatin concentration: 10%.

Firstly, we explored the influence of the applied pressure in shear wave speed. Shear wave signals measured with TWE method at different applied pressures phantom-sensor and with the same frequency excitation (300 Hz) and gelatin concentration (10%) are shown in Figure 7.

**Figure 8:**
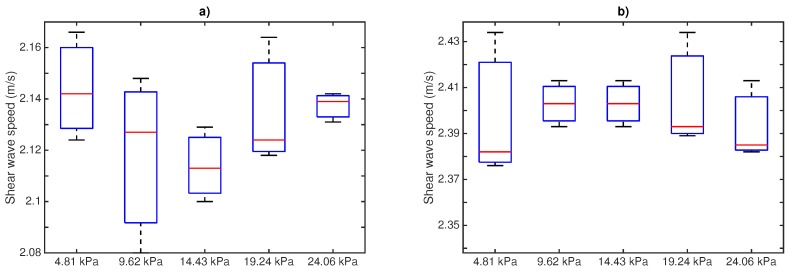
Box and whisker plots of shear wave speed measurements at different applied pressures phantom-sensor. Mean (lines within boxes), interquartile range (IQR, boxes) and extreme values (whiskers) are shown. (**a**) 8% gelatin; (**b**) 10% gelatin.

The shear wave speeds calculated from measurements using the proposed time of flight method are shown in Figure 8. Box and whisker plots are represented for the designed phantoms at 8 and 10% gelatin concentration (three samples per concentration), 300 Hz frequency and for the five different pressures. It can be extracted from the plots that there is no correlation between the applied pressure and the shear wave speed.

**Figure 9:**
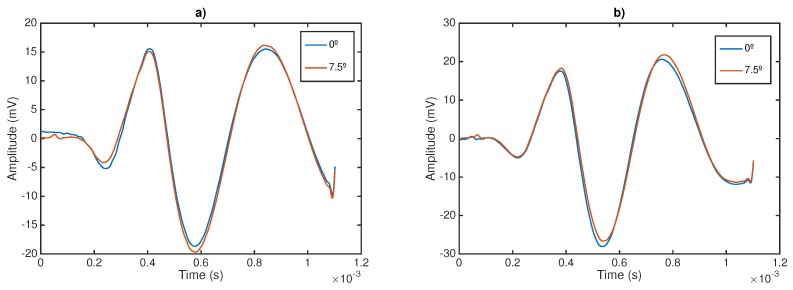
Measurement of shear wave speed at different angles of incidence sensor–phantom; frequency: 300 Hz. (**a**) 8% gelatin; (**b**) 10% gelatin.

Secondly, to assess the other outcome of the sensor sensitivity study, two shear wave signals have been plotted at two different angles of incidence: 0 and 7.5${}^{\circ }$ (see Figure 9).

**Figure 10:**
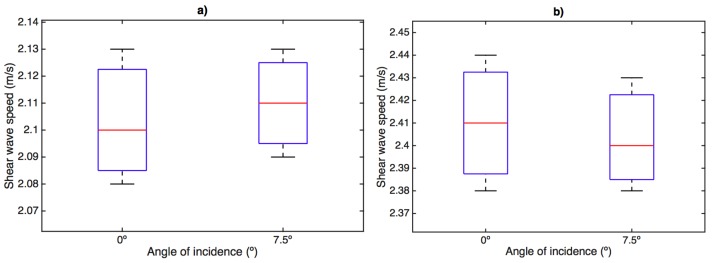
Box and whisker plots of shear wave speed measurements at different angles of incidence phantom–sensor. Mean (lines within boxes), interquartile range (IQR, boxes) and extreme values (whiskers) are shown; frequency: 300 Hz. (**a**) 8% gelatin; (**b**) 10% gelatin.

Shear wave speed measurements are represented in Figure 10. Box and whisker plots are shown for the three designed phantoms at 8% and 10% gelatin concentration, 300 Hz frequency and for the two different angles of incidence. The same conclusion is extracted when varying the angle of incidence. No significant variations were obtained.

**Figure 11:**
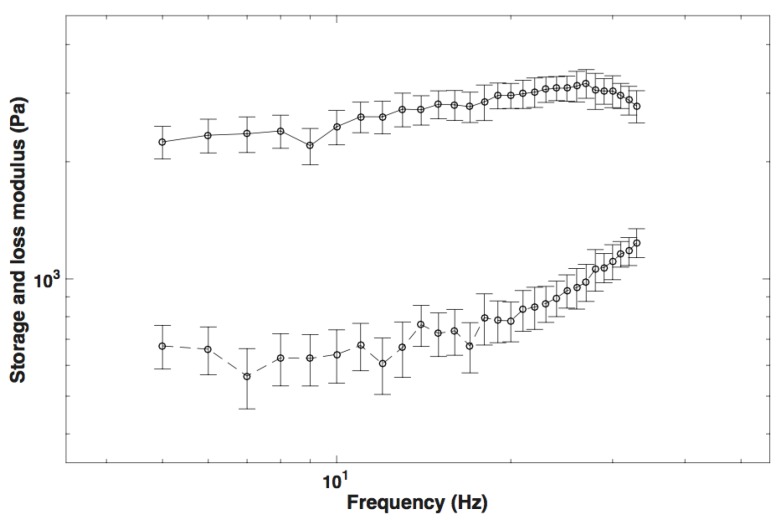
The storage and loss shear modulus measured by rheometry in cervical tissue.

The real and the imaginary part of the complex shear modulus measured in cervical tissue by rheometry are shown in Figure 11. The measurements were taken three times for each cervix to obtain the mean and the standard deviation. 

**Figure 12:**
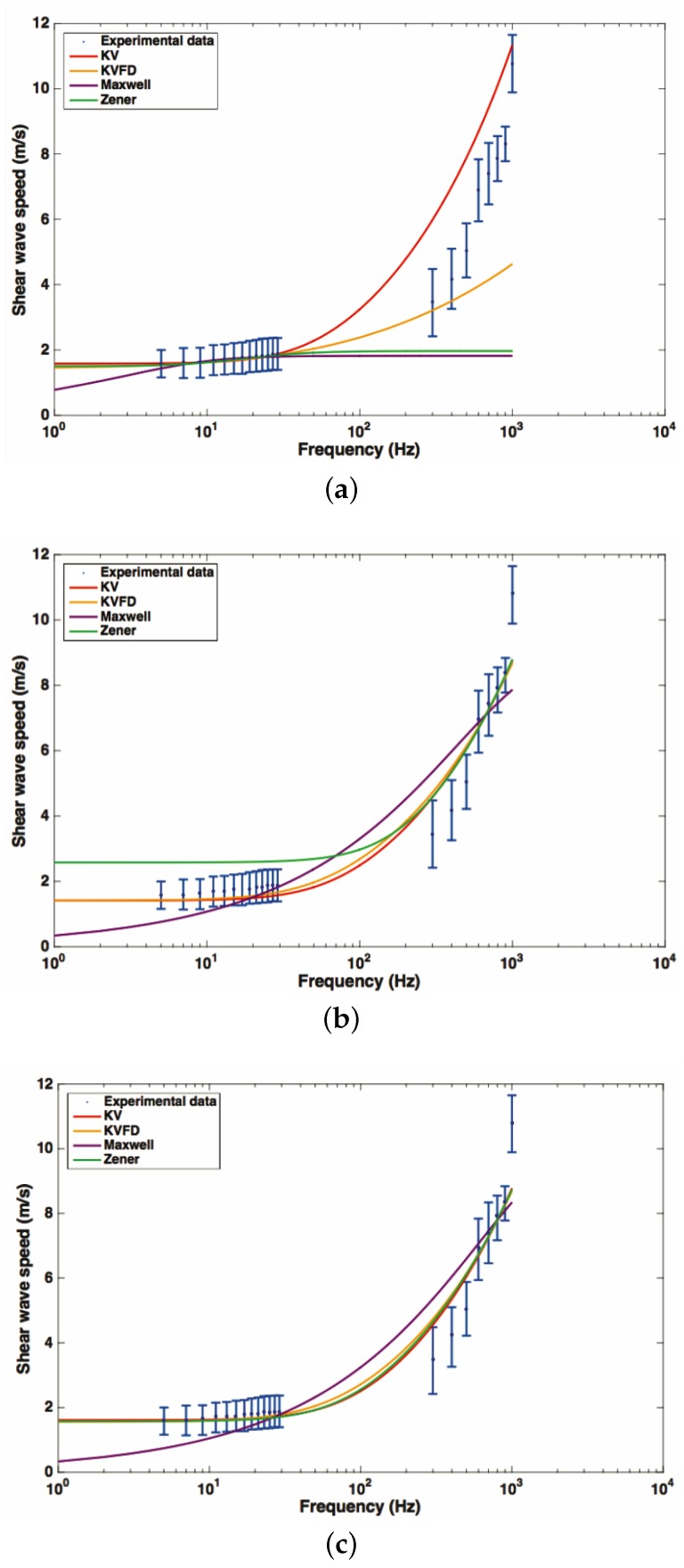
Fitted curves using data from rheometry, from elastography and using the combined data from the rheometry and elastography for each model. The circles are the mean values over the three cervix and the horizontal bars are the standard deviations. The curves for the Kelvin–Voigt (solid red line), Kelvin–Voigt Fractional Derivative (solid yellow line), Maxwell (solid purple line) and Zener model (solid green line) are shown. (**a**) data from rheometry; (**b**) data from elastography; (**c**) data from rheometry and elastography.

Figure 12 shows the fitted curves using data from rheometry, elastography and both sources of data simultaneously, for each rheological model. Frequencies for rheometry measurements ranged from 4 to 30 Hz, while, for the elastography technique, there was a wider range from 300 to 1000 Hz.

Table 2 and Table 3 show the viscoelastic parameters using the data from rheometry, elastography and a combination of the two methods for Kelvin–Voigt and Kelvin–Voigt Fractional Derivative models. The high-frequency components of the dispersive curve play a much larger role than the low-frequency components. The curve shape or dispersive pattern is primarily determined by the high frequency components. KV and KVFD models matched satisfactorily all the data from the high to the low-frequency regime. The findings can also be confirmed by the results of Table 2 and Table 3, showing that the elasticity and viscosity values obtained from the high-frequency TWE data were very similar those from the overall rheometry + TWE data, although they differ from those obtained from the low-frequency rheometry data.

## 4. Discussion

Characterization of the viscoelastic properties of soft tissues is a key step for the development in many medical applications based on elastography imaging. In the case of cervical tissue, a special interest lays on the study of the evolution of the viscoelastic properties during pregnancy, in addition to their correlation with the gestational age. Little data for the case of viscoelasticity of human cervical tissue can be found in the literature [26].

The increasing interest in elastography techniques for measuring viscoelastic parameters is demanding appropriate validation studies. Currently, the traditional standard for evaluating the viscoelastic properties of soft tissues is based on rheological methods. However, these techniques are limited to in vitro and ex vivo samples. In addition, whereas the trustful range of frequency in the rheological test is from the quasi-static regime to no more than 50 Hz, dynamic elastography techniques usually use a range from 100 Hz to 500 Hz. This discrepancy makes the comparison process a challenge. However, due to the lack of alternatives, the validation between both techniques has been accepted in previous studies [44,45].

The presented TWE method is based on the transmission and detection of shear waves. Shear waves travel axisymmetrically from the center of the probe, where the mechanical actuator is placed, towards the outer side where the piezoceramic receivers are located. In this study, the TWE technique was proved capable of successful transmitting and receiving shear waves from 300 Hz to 1 kHz. This range of frequencies represents an obvious advantage when compared with commercial elastography devices [7], since a broader range of frequencies provides more significant information about the viscoelastic behavior of the tissue, in particular for the viscous component, which is more sensitive to high frequencies [44].

Here, a study with respect of two variables involved during the testing procedure was presented: the normal pressure applied and the angle of incidence between the probe and the normal direction of the tissue surface. This simplified study may fall into a preliminary analysis of the intra-operator dependency of the method. The range of pressures (ranging from 4.81 kPa to 24.06 kPa) and angles (0${}^{\circ }$ and 7${}^{\circ }$) were chosen in close relation with those experienced during a normal testing procedure. Gelatine phantoms with 8% and 10% concentration (*w*/*w*) were selected as an adequate representation of the viscoelastic behavior of soft tissues. As expected, higher concentration of gelatine produced higher shear wave speed due to higher stiffness. Variable levels of pressure produced a minimal variation in the shape of the collected signal, as can be seen in Figure 7. The standard deviation values of shear wave speed varied between 0.018 to 0.031 m/s, including all measurements for the range of frequencies studied (300–1000 Hz). The impact of this variation on the reconstructed values of shear wave speed was non-significant for all the frequencies. Therefore, no correlation between the level of pressure and shear wave speed was found. The same effects were observed when varying the angle of incidence. Figure 9 shows hardly noticeable variations in the measured signals. These observations were found consistent all over the different gelatine concentration phantoms tested, which seems to indicate that the TWE technique is, at least in gelatine-based phantoms, a robust and low user-dependent elastography method. However, as observed in the experiments, simple gelatine-based phantoms shows a low viscous behaviour, which is not proper of soft tissues. This low viscous behavior might be responsible for such stable results against the two variables of the user-dependency test. Different recipes for obtaining viscous elastography phantoms have been published, for instance by adding castor oil to the gelatine-based original recipe [46]. In further studies, elastography phantoms with higher viscosity will be tested. These analysis must be also extended to tissues exhibiting characteristics more similar to biological tissues, which may not be homogeneous [4] to define the validity limits of these studies and to develop clinical practice protocols to correct deviations when using the designed device.

After the positive outcomes of the sensitivity analysis, a series of experiments were performed on ex vivo human cervical tissue. The experiments aimed to combine information from rheometry and TWE providing two sources of data to find the most suitable rheological model to fit the cervical tissue behavior.

Figure 12 shows the dispersion curves of shear wave speed regarding the frequency. Since both rheometry and TWE techniques obtain shear wave speed values at different ranges of frequency, rheological models are required to combine the dispersive data. In this study, the classical models of Maxwell, Kelvin–Voigt, Zener and Kelvin–Voigt Fractional Derivative were employed to fit the dispersive data. As proposed by Lin et al. [44], three types of fitted dispersion curves were obtained depending the data: (a) by using only the data from low-frequency regime generated by rheometry, (b) by using only the data from the high-frequency data provided by TWE, and (c) by fitting the data from both low and high-frequency ranges.

Whereas the fitted curves obtained by using the TWE data were overall in good agreement with the data from rheometry (Figure 12b), the curves using the rheometry data diverged from those using TWE (Figure 12a). Additionally, the dispersion curve after fitting the whole set of data, this is rheometry + TWE (Figure 12c), practically coincided with the curve generated by using only the TWE data. These findings agree with the observations by Lin et al. [44], and point out the relevancy of the viscous effects at higher frequencies. Furthermore, it uncovers the difficulty of characterizing the viscous effects of soft tissue by limiting the experiments to classical rheometry techniques.

When comparing the four rheological models, it can be observed that the Maxwell model showed difficulties to represent the dispersion of shear wave speed along the whole range of frequency. The Zener model adjusted the data successfully when considering both the rheometry and the TWE outcomes. However, when fitting only data from TWE, it showed some divergence at the low-frequency range.

On the other hand, Kelvin–Voigt and its fractional derivative version matched all the data satisfactorily from the high to the low-frequency regime. The fact that both models produced practically the same dispersive curve can be understood by observing Table 2 and Table 3. The values obtained for the α parameters of the Kelvin–Voigt Fractional Derivative model were close to 1, which transforms the model directly into a classical Kelvin–Voigt. A two-parameter model as the Kelvin–Voigt usually will be preferred against a three-parameter model as the fractional version, due to practicality and faster computation. Further studies must be performed to analyze the consistency of all the findings above shown.

## 5. Conclusions

A TWE technique for characterizing the viscoelastic properties of soft tissue, and, in particular cervical tissue, is proposed. The shear wave curve dispersions for cervical tissue produced by the TWE method were in good agreement with the data obtained from rheometry. However, these results from rheometry were not able to reproduce the viscous effect on the speed dispersion at high frequencies. Further studies must be carried out to analyze the consistency of these observations. The Kelvin–Voigt model and its fractional derivative version fit the cervix experimental data along the whole range of frequency successfully, and this is considering rheometry and TWE.

The results obtained in this study pave the way to carry out a cross sectional study, which aims the correlation between the gestational age and the viscoelastic properties of the cervix during pregnancy.

## Figures and Tables

**Table 1:** Validation analytic design vs. FEM.

Disc Frequency [Hz]	2.80 $\times \;10^{4}$
Ring Frequency [Hz]	2.82 $\times \;10^{4}$
FEM Frequency [Hz]	2.82 $\times \;10^{4}$
Error 1 (%)	0.68
Error 2 (%)	0.04

**Table 2:** Viscoelastic parameters using the data from rheometry, TWE, and a combination of the two methods for the Kelvin–Voigt model.

Cervix Number	Elasticity μ (kPa)			Viscosity η (Pa·s)		
	Rheometry	TWE	R + TWE	Rheometry	TWE	R + TWE
1	1.69	2.13	1.82	5.31	4.32	4.21
2	1.83	2.52	2.10	6.52	4.55	4.64
3	1.85	2.64	1.84	7.19	4.9	4.65
Mean	1.79	2.43	1.92	6.34	4.59	4.5
Standard Deviation	0.08	0.26	0.15	0.95	0.29	0.25

**Table 3:** Viscoelastic parameters using the data from rheometry, TWE, and a combination of the two methods for the Kelvin–Voigt Fractional Derivative model.

Cervix Number	Elasticity μ (kPa)			Viscosity η (Pa·s)			Fract. Deriv. Power α		
	Rheometry	TWE	R + TWE	Rheometry	TWE	R + TWE	Rheometry	TWE	R + TWE
1	1.10	2.13	2.07	12	4.02	4.54	0.42	0.98	0.99
2	0.80	1.93	2.22	31	4.21	4.73	0.13	0.99	0.96
3	0.86	2.12	1.74	26	4.46	4.65	0.20	0.94	0.99
Mean	0.92	2.06	2.01	23	4.23	4.64	0.25	0.97	0.98
Standard Deviation	0.15	0.11	0.24	9.84	0.22	0.09	0.15	0.02	0.01

## Abbreviations

The following abbreviations are used in this manuscript:

SE	Static elastography
DE	Dynamic elastography
TWE	Torsional wave elastography
3DMMRE	3D multifrequency MR elastography
FEM	Finite Element Models
FEAP	Finite Element Analysis Program
M	Maxwell rheological model
GM	Generalized Maxwell rheological model
KV	Kelvin–Voigt rheological model
KVFD	Kelvin–Voigt Fractional Derivative rheological model
Z	Zener rheological model
ERDF	The European Regional Development Fund
